# Book Reviews

**Published:** 1826-01

**Authors:** 


### CRITICAL ANALYSIS
OF
### ENGLISH AND FOREIGN LITERATURE,
RELATIVE TO THE VARIOUS BRANCHES OF
### Medical Science.

Quæ laudanda forent, et quæ culpanda, vicissim

Illa, prius, cretâ; mox hæc, carbone, *notamus*.—PERSIUS.

### DIVISION I.

### ENGLISH.

**Art. I.**—*On the Action of Calomel. Being a Critical Analysis of Part III. of "Sketches of the most prevalent Diseases of India; comprising a Treatise on the Epidemic Cholera of the East, &c."* By James Annesley, Esq. &c.—8vo. pp. 464. London: T. and G. Underwood. 1825.

Having, on a recent occasion,* entered somewhat largely into the subject of the Indian cholera, we do not feel disposed to return to the discussion; particularly as we believe that the great majority of our readers are more interested in what occurs under their own immediate observation, than in maladies, however dreadful, of which they only hear as the scourges of distant countries. Influenced by these considerations alone, and in no degree from regarding the work before us as inferior in interest to others which have preceded it, (for this is very far from being the case,) we have limited our analysis to that part of the subject which possesses general interest: we mean the practical observations on the effects of Calomel.

* See our analysis of a "Report of the Epidemic Cholera," &c. by W. Scot; No. for Jan. 1825.

It is truly remarked by our author, that, although this medicine has been employed in practice during the last two centuries, yet even now it seems to be matter of doubt to what extent it may be given with advantage, and what its *modus operandi* on the living system may be. At its first introduction, it was chiefly had recourse to in doses of a scruple and upwards, as an active purgative, either alone or in combination, in those cases where a full and free evacuation of the secretions of the liver and alimentary canal were intended, as well as to effect the restoration of any natural secretion which had been arrested by disease. This is precisely the application of calomel which has been proposed by various writers of the present day, who have been supposed to originate a method which, in fact, they only attempt to revive. A short quotation from Mr. ANNESLEY's volume will set this matter at rest.

"Horstius stated that the 'mercurius dulcificatus' may be given in doses of one scruple, or half a drachm, 'ad viscidos humores magis attenuandos;' and Deleboe Sylvius recommended it in the same doses, as an attenuating purgative. Wepfer prescribed it in doses of twenty grains, in combination with other purgatives, in affections of the head. Dr. Friend advised it in simple doses, in conjunction with purgatives and emmenagogues, in emansio and obstructio mensium. Schroder has expressly stated that 'mercurius dulcis vulgaris, draco mitigatus, omnes humores noxios, sine perturbatione laude expurgat, unde vel infantibus exhiberi poterit. Dari potest ad ℈ss.: cum aliis purgantibus à gr. viij. ad xv. et plures.' Juncker has made mention of the exhibition of calomel in doses of twenty and thirty grains; and Geoffroy has recommended it to be given in doses of from five grains to thirty, according to the circumstances of the case. These authors seem, however, not ignorant of the mode of exhibiting this preparation in much smaller doses; but, whenever they resorted to this manner of prescribing it, their object appears to have been to affect the system with the mercurial action. Whenever they were desirous of obtaining advantageously its purgative effects, they exhibited it in the large doses above stated, at considerable intervals between each; a mode of exhibiting it founded on their experience of its effects." (P. 378—380.)

After this period, however, the remedy was employed with considerably more caution,—or, perhaps, we might say, timidity; and, according to our author, with considerably greater risk of producing the deleterious consequences which they feared from its too free administration: "two grains were found to irritate,—five were therefore inferred to irritate more." This, unquestionably, is the general impression on the minds of practitioners in this country, with respect to the agency of calomel: distrustful of its effects in large doses, they endeavour to remove all risk of inconvenience by giving it in very limited portions, while they attempt to secure all the benefit of its operation by the frequency of its repetition. Many practitioners, in an acute case, would administer three grains of calomel every three hours, who would not venture to give a scruple once in twenty-four hours. This we assert as matter of fact,—not as expressing any opinion touching the comparative merits of the two methods: but we may mention that Mr. Annesley has distinctly made up his mind between them, and thinks scruple doses once a-day more powerful in arresting the progress of disease, and less apt to be followed by serious constitutional evils. Hear him:

"The prejudices of early instruction are difficult to be overcome: they lead us to draw conclusions from inadequate premises, and sometimes to reject useful inferences, because they accord not with preconceived or generally adopted opinions. Ignorance also leads us to adopt, without examination, what is generally received, even although the most palpable objections may be found to it in our daily experience.

"Influenced, in some degree, by a similar partiality of judgment and indolence in research which I here condemn, I was in the habit of administering calomel only in moderate doses, until I perused the valuable work of Dr. Johnson; after which I began, in my hospital at Trichinopoly, to exhibit it in doses of one scruple each, to a patient in the advanced stage of dysentery: its action in this instance was so strikingly useful in procuring ease and comfort to the patient, that, although the case was not successful, I determined to give it a trial at the commencement of those acute diseases which we find most distressing and destructive in India,—namely, in dysentery, hepatitis, and fever; diseases which, in general, commence with great excitement, and excessive irritability of stomach. I accordingly adopted this practice, and have followed it for upwards of eight years, and in no instance have I had reason to be dissatisfied with its effects.

"Having been even at that time prepossessed in favour of large doses of calomel, it was not a difficult matter to make me a convert to the practice; but I adopted it with very different views from those with which it was then recommended, and modified it accordingly, as will be seen in the sequel: nevertheless, so great were the prejudices that existed against this practice, even amongst men of professional eminence and reputation, that I have often doubted my own judgment in suggesting to those gentlemen who were placed under me, on their first arrival in India, the propriety of administering calomel in larger doses than are commonly thought necessary, although the result of my own experience was so decidedly in favour of the practice; and I have sometimes felt great difficulty in meeting, and successfully resisting, the various objections which have been made to it. I consequently did not press its use, but gave the confident assurance that calomel could be used in large doses with perfect safety; and established the fact by showing its effects when so administered by me. A very short time convinced them of its advantages, and the practice became general from conviction, and not from persuasion." (P. 383—385.)

The author deprecates, and in our opinion not without reason, the idea so often inculcated, that mercury, to be useful, must affect the gums,—nay, that this affection is to be regarded, not merely as an index, but in some degree as a measure, of its utility. Were this the case, how useless would calomel be in the fevers of this country; and we put it as a question to our readers, whether they have *ever* seen salivation produced in common continued fever, by the administration of free doses of calomel every night, *unless the exhibition of the medicine was continued after the fever had given way?* We believe that it is a very bad rule to continue the administration of calomel till salivation be produced; for in some this never will occur: but that, on the contrary, it is a good rule to intermit the mercury as soon as the gums are at all affected, as the constitution may then be easily kept under its influence. In such cases, however, we suspect that the gums become affected because the fever has subsided,—not that the fever has subsided because the gums have become affected.

With a view of ascertaining the effects of calomel upon the mucous membrane of the stomach and bowels, the author undertook some experiments upon dogs; of which the following interesting details are given:—

"About eleven o'clock A.M. Dec. 1, 1823, I gave three healthy dogs the following doses of calomel,—viz. ʒj. ʒij. and ʒiij. After they had taken the calomel, they were kept in a room, and observed narrowly for twenty-four hours.

"The dog who took ʒj. did not appear to feel any kind of sickness till night, when he vomited a little. He was lively the whole time, and ate his food well; had been purged two or three times: dejections very dark grey colour.

"The dog who took ʒij. was likewise lively, and ate his food well: vomited two or three times, and was purged more than the other. He passed tape-worms, and the dejections were black.

"The dog that took ʒiij. was heavy, and apparently uncomfortable the whole day, but did not vomit at all. He was purged, and passed a very long tape-worm: dejections also black. Although he looked somewhat heavy before he took the calomel, and was apparently dull and uncomfortable during the day on which the calomel was administered, he improved very much in his appearance on the following day, and was very lively.

"At ten o'clock A.M. on the 2d December, (twenty-four hours from the time at which the calomel was taken,) the three dogs were hanged; and, as the largest dose was given with a view of ascertaining the worst effects of this preparation, I first examined the dog that took it, five minutes after he was dead.

"The veins were beautifully injected, the liver healthy, and the gall-bladder *full of bile.*

"The external coat of the stomach was of a pale colour, and seemed to be rather thickened.

"The small intestines had a peculiarly thickened feel, very similar to what is observed in cases of cholera; but I am not quite sure whether this thickened state is not natural to the healthy intestine of the dog.

"The stomach was laid open: its internal surface was considerably corrugated, and of a dusky red colour, but possessing neither the appearance of high arterial action or of venous congestion. The corrugations were in a longitudinal, and not in a circular direction.

"The small intestines were laid open: their internal surface was loaded with thick, tenacious, cream-coloured matter, such as is generally found in the intestines of those who die of cholera. It appeared that the calomel, in this instance, had no other effect upon the dog than that of diminishing the vascularity of the stomach, as it did not seem to have mixed at all with the secreted matter of the intestines, or to have acted upon the gall-bladder. Probably the time was not sufficient for the purpose.

"The dog which was next opened was that which took ʒij. of calomel.

"The appearance of the stomach, both externally and internally, was infinitely more vascular than observed in the preceding experiment. The corrugations were, however, precisely of the same nature as those already described, and the venous system was beautifully injected; but there was a very considerable flow of bile in this dog, and the contents of the duodenum were more fluid, and less tenacious.

"The dog who took ʒj. was last opened, and in him also the venous system was highly injected; but we were surprised to see a much higher degree of vascularity in the stomach of this dog, particularly at the internal surface, than in either of the two others.

"The bile, too, had flowed freely into the duodenum, and the contents of the bowel were highly coloured with bile, and not at all tenacious. The corrugations were of the same character, viz. longitudinal.

"Observing that the vascularity of the stomach was greatest in the dog which took the smallest quantity of calomel, I procured a healthy dog, and, without giving him any of this preparation, had him hanged, and examined five minutes after he was dead, in order to see the natural state of the stomach,—at least unoperated upon, and unchanged by any medicine. I was greatly surprised to find that the stomach of this dog was infinitely more vascular than that of either of the three dogs already examined, and was in what I really would have considered a high state of inflammation. The corrugations were circular, and more or less vascularity extended throughout the alimentary canal, which was covered with a glairy transparent mucus.

"In order further to ascertain the correctness of these inferences, the following experiments were performed.

"At one o'clock A.M. December the 6th, 1823, I gave ʒiij. of calomel to two dogs each, that had been kept in a separate room for two or three days previously, and had been fed upon rice and sheep's head. After taking the calomel, they were sick, and vomited, but seemed to suffer no other inconvenience; for they ate well, and were seemingly in good spirits.

"At eleven A.M. the 8th December, forty-eight hours after taking the calomel, one of the dogs was hanged: the other was preserved, to show that no inconvenience attended the ingestion of so large a dose of calomel; and this was amply proved, as the dog continued well, and was in better condition a month after the experiment than he was before.

"I examined the dog that was hanged as soon as possible after he was dead, and the following were the appearances exhibited:

"The veins, as usual, were beautifully injected; the liver was quite sound, and the gall-bladder rather empty. The external appearance of the stomach, which was very much distended with rice, was of a pale colour, with some large blood-vessels spread over it; and, on being laid open, the rice near the pylorus was marked with bile, of a beautifully bright orange colour, while that in the upper part, near the cardia, was perfectly unchanged. On removing the rice from the stomach, it was found that its surface only was tinged with bile.

"The stomach, cleared of the rice, exhibited a beautifully corrugated appearance throughout its whole surface, with a peculiarly pale pink blush; but nothing like excitement or vascularity was evident.

"The corrugations were in the transverse direction, and were more marked near the cardia; but were less so at the lower part of the stomach, near the pylorus, where it was deeply tinged with healthy bile. The whole surface of the duodenum was covered with bile, without showing the slightest trace of vascularity or viscid secretion.

'The jejunum was filled with digested food, well mixed with bile. In the lower part of the intestine, the contents were more consistent, and of that peculiar blackish grey colour which is always produced by mixing calomel with the secretion of the intestine.

"The inner surface of the colon was in a high state of arterial vascularity; the transverse ridges being not unlike the valvulæ conniventes in the human small intestine.

"The rectum was likewise in an increased state of vascularity, with longitudinal corrugations.

"From comparing the state of this dog's stomach with those which I first examined, it seems that the first effect of calomel, in large doses, is not only to diminish vascularity, but also to produce a peculiar action of the fibres of the stomach, and that this organ requires a certain period to elapse before it can resume its natural function." (P. 386—396.)

Mr. Annesley, in the next section, proceeds to state his belief that calomel produces a chemical action upon the mucous secretions, in consequence of which their mechanical properties become modified to a great extent. He has frequently mixed, *in situ*, small quantities of calomel with the tenacious mucus occasionally found in the intestinal canal, on examination after death: the secretion, he informs us, when thus treated, assumes a dark grey colour,—becomes more fluid, less tenacious, and consequently more easy to be detached from the subjacent membrane.

He conjectures that a partial decomposition of the calomel is effected; a portion of the mercury being converted into a dark grey oxide, and imparting its colour to the secretions with which it is mixed. If then, it is asked, the calomel thus attenuates and separates the viscid mucus, may not its operation in the duodenum be the means of removing obstructions from the common duct, caused by this secretion; thus effecting a discharge of bile, which has been mechanically retained? "In this case the dose of calomel may be considered as acting chemically upon the mucous secretion, and mechanically as regards the duct." It is considered of importance that the ducts should be cleared in the first instance; as it is argued, that increasing the quantity of bile, by small doses of calomel, before the ducts were sufficiently cleared for its free exit, would but augment the evil. Hence the necessity of giving purgatives until dark green motions have been produced. "When, therefore, we see the change from dark grey (the colour which calomel alone gives the mucous secretion,) to dark green, we may rest satisfied that the ducts are emulged, and that the calomel and cystic bile are acting conjointly upon the bowels. Hence the propriety of continuing this remedy till healthy action be produced, will appear evident."

A general idea of the author's manner of employing the remedy, may be gathered from the following observations:—

"Section IV.—*General Remarks on the Use of Calomel in Disease.*

"It is not the intention of these observations to recommend the indiscriminate use of calomel; but I maintain, from very extensive experience of its effects, and from the experiments already stated, that in many acute diseases, and particularly in those of India, it may be given boldly, and without risk; and that injurious effects are more likely to be produced by frequently-repeated small doses, which keep up a certain degree of irritation and nausea, than by a full dose given at once, and discontinued when the objects looked for are gained. These objects I conceive to be to allay irritability, diminish vascular action, and to *cleanse* the intestinal canal of the tenacious matter which often lines it, and in many cases almost completely obstructs the passage through it.

"In these diseases, therefore, in which we have reason to suppose, from the great irritability of the stomach, the state of the tongue, and the functions of the abdominal viscera, that increased vascularity of the digestive canal is present, with a deranged state and accumulation of the mucous and other abdominal secretions,—as in all the types of fever, dysentery, liver complaints, &c.—calomel, in doses of from ten to twenty grains, either alone or variously combined, according to the circumstances of the case, is an excellent remedy.

"In these diseases, I have been generally in the practice of giving, at bed-time, twenty grains of calomel, with one or two grains of opium, and sometimes without the opium, and a smart purgative draught the following morning. This practice I have repeated daily, until the excretions assumed a healthy hue. A tonic laxative was then exhibited, and continued till the natural functions of the bowels were completely restored. In these diseases, I never wished to see the mouth in the least degree affected: whenever this happened, I considered the salutary effects of calomel interrupted, because its use must be then discontinued; and it was my object to act upon the secretions of the intestines, to diminish vascular excitement in the intestinal canal, and not, in the most remote degree, to act upon the salivary glands.

"It is evident, from the preceding, that calomel is not a suitable medicine in those cases wherein an opposite state of the digestive canal and of the secretions to that described exists, and that it is calculated to prove injurious under such circumstances. Judgment, and an experienced discrimination, are requisite to the beneficial administration of this remedy under most circumstances; and I consider that it is chiefly owing to the want of this, that it is so often used with little advantage, and that so much diversity of opinion exists amongst medical men respecting the administration of it in various diseases.

"Although I was for many years fully aware of its effects in allaying irritation of the stomach, and in procuring abundant discharges of tenacious and morbid matters from the bowels, yet it is comparatively much more recently that I was led to analyse more closely its effects in disease. I always observed that the frequent repetition of small doses of calomel generally produced nausea and uncomfortable feelings, and completely deranged the functions of the stomach, which was not the case with full doses of this preparation, when given at considerable intervals, and in such a manner as to act decidedly upon the secretions and functions of the bowels; and more particularly when aided by a purgative on the following morning.

"When it is desirable to affect the constitution with the mercurial action, and to keep up that action, in order to remove any glandular obstruction, I cannot advise this object to be attempted by means of the exhibition of small doses of calomel, given at short intervals. I have always found this practice attended with unpleasant effects, and the digestive organs not unfrequently injured by it. I therefore recommend the blue-pill in preference; and that the use of it, even, should not be pushed further at any time than the production of a slight soreness of the mouth, and even not so far as that, if decided benefit follow the exhibition of the remedy, short of this effect." (P. 405—409.)

Mr. Annesley then proceeds to consider the application of calomel to particular diseases; and through these we shall follow him seriatim.

### *Of the Employment of Calomel in the Treatment of Intermittent, Remittent, and Continued Fevers.*

Three indications are mentioned as capable of being fulfilled by its administration in these diseases:—viz. first, a diminution of the irritability of the stomach, particularly if it depend upon increased vascular action of the inner membrane of this viscus; secondly, a correction and discharge of the secretions of the internal surface of the alimentary canal and large secreting organs; and, thirdly, increasing the action of these great secreting organs, and exciting the functions of the vascular system in general,—without, however, affecting the salivary glands.

In *intermittents*, the author prescribes scruple doses, which are given at bed-time, with a view of allowing it to operate undisturbed till next morning, when free evacuations are produced by infusion of senna, with salts and tartarised antimony. Occasionally an emetic is necessary; in which case, the author recommends it to be taken in the forenoon, and the dose of calomel already mentioned exhibited at bed-time. On the second night, a similar treatment is pursued; or ten grains of calomel may be given, with five or six of aloes. As soon as the tongue begins to clean, and the excretions assume a healthier character, the cinchona, combined with aromatics, may be given in divided doses shortly before the expected paroxysm. But we are cautioned not to begin the bark until hepatic bile is seen in the excretions; and particularly to avoid giving it whilst "pain, tenderness, fulness, and oppression, are felt in the hypochondriac and epigastric regions." This advice is unquestionably correct: at the same time, from the manner in which it is given, one would be led to suppose that practitioners in this country had administered bark under the circumstances pointed out as contra-indicating it. If this be Mr. Annesley's impression, he may rest assured that he is mistaken; and the only point of his practice in agues, about which difference of opinion is likely to exist, relates to the dose of calomel: he gives twenty grains where, in this country, one fourth of that quantity is generally given. As soon as the disorder yields, calomel is administered in much smaller doses; or, what is to be preferred, the blue-pill, with myrrh and aloes. When great irritability of the stomach is present during the paroxysm of an ague, scruple doses of calomel, with one or two grains of opium, may be given.

In *remittent* fevers, the author first endeavours to relieve the most urgent symptoms by bleeding; after which an emetic, or a full dose of calomel, is exhibited, according to circumstances. If there be foulness of the tongue and bitter taste in the mouth, the emetic is given first, and the calomel afterwards, as in the case of agues. Next morning, purgatives (and, if these fail, cathartic glysters,) are administered; a saline mixture, with the spirit ætheris nit. and tartarised antimony, being continued during the day. Congestions in the viscera are watched, and promptly relieved by local bleeding. And this plan is persevered in till the fever assumes more of a regular intermittent form; after which, an alterative and laxative course of medicine is pursued, with the addition of cinchona. Mr. Annesley is of opinion that there is a connexion between these fevers and certain states of the moon (as the full and change), and, when it has been ascertained "at what time of the moon's age the paroxysms supervene, the bark is given two or three days previous to that, and continued until the period has elapsed."

The *continued* fevers which the author has met with are decidedly of an inflammatory character in their first stage, and calomel is exhibited, although, it is to be remembered, exclusively with a view to the two first indications above mentioned: indeed, as already stated, during the inflammatory stage of fever, there is little risk of the calomel inducing its specific effects. Here, as in other cases where there is much irritability of the stomach, opium is to be continued with the calomel; and, if there be any appearance of spasmodic constriction about the gall-ducts, this may be given to the extent of two or three grains. This addition of opium, we are assured, has no tendency to prevent the purgative effects of calomel; but, on the contrary, rather promotes its laxative operation.

### *Of the Use of Calomel in the acute Diseases of the Liver and Gall-bladder.*

Mr. Annesley states it as his opinion that calomel ought to be used in these complaints with a view to its purgative effects alone, and "never in such a manner as may give rise to its absorption, and consequent constitutional effects:" and he continues—"I am anxious, in acute affections of the biliary organs, to avoid the constitutional effects of calomel, because I believe that, when these are produced, the energies and vital resistance of the system are thereby impaired, and the presence of this mineral in the circulation tends to keep up, in the inflamed part, a degree of excitement and irritative action which would otherwise subside; and which, I am persuaded, tends in many instances, when allowed to proceed, to occasion chronic derangements of this viscus, and even to give rise to abscesses, if the inflammation be seated in the glandular structure of the organ." (P. 424.)

These observations apply equally to inflammation of the gall-bladder and ducts.

### *On the Use of Calomel in chronic Disorders of the Biliary Organs.*

The simplest form of chronic disorder of the liver is stated by the author to be imperfect discharge of its functions: this, however, may lead to acute and chronic hepatitis, particularly the latter. This imperfect discharge of the functions of the liver is frequently dependent upon debility of the general constitution, and may be treated accordingly; calomel being used occasionally as a purgative: "but, in this form of disorder, mercurials ought not to be given so as to occasion ptyalism."

When, however, this simple state of deranged function continues for a certain time without amendment, it gives rise to congestion in the portal veins and infarction of the biliary ducts. This more severe form of functional disorder is marked by muddiness or yellowness of the countenance, and heaviness of the eye; by pale or clay-coloured motions; by oppression and fulness, but not pain, about the epigastrium; and by the patient becoming weak and emaciated in body, and depressed in mind. Under such circumstances, we are advised to begin with calomel, either alone or combined with aloes, over night, and a black dose next morning; which remedies are to be continued until the stools assume a healthy appearance, when the blue-pill and aloes are to be given at night, and laxatives with tonics during the day. If these fail, the nitro-muriatic acid bath is recommended, with blistering to the region of the liver.

The following account of the consequences of these derangements, if not effectually remedied, are worthy of attention.

"The derangement now described may be neglected, or it may be partially removed; in which case it generally returns. In either instance it will terminate, in a longer or shorter time, according to circumstances, in more serious disorder. It will give rise to vascular action, of a sub-acute kind, in the substance of the liver, which, whilst tending to overcome the obstruction previously existing, also gives rise to the effusion of lymph in the structure of the part where such re-action supervenes. Thus, enlargements of parts, or of the whole of the liver, take place; or the formation of tubercles and schirrous hardness is the result, even although the patient may not have been the subject of previous acute disease of this viscus. It ought, however, to be remarked that this, as well as the other chronic derangements of the biliary organs, are often the result of a previous attack or attacks of acute hepatitis: but, whether occurring as the sequelæ of the acute form of the disease, or as the primary disorder, either in a patient who has enjoyed previous good health, or in one who has suffered under some other disorder, as intermittent or remittent fever, &c., the symptoms and the treatment will be nearly the same in most of their important constituents.

"The form of chronic disorder now under consideration is indicated by the presence of the greater number of the symptoms already detailed, with the addition of a dull pain under the blade or top of the right shoulder, with uneasiness, and occasional pain and fulness, in the region of the liver, or at the epigastrium; with a white or foul tongue, dry harsh state of the skin, occasional slight paroxysms of fever, &c. This form of disorder requires the exhibition of calomel in the manner already pointed out, in conjunction with local depletions, blistering on the region of the liver, warm poulticing, and the nitro-muriatic acid bath. In this form of disorder, the alternate use of large doses of calomel and the cathartic draught is required for a longer period, and the subsequent employment of aperients and laxatives should also be longer persisted in. More caution is requisite in resorting to the exhibition of tonics, and these should never be prescribed uncombined with aperients or laxatives.

"In addition to the congestion of the portal veins, with a loaded state of the biliary canals, and sub-acute action in parts of the viscus now alluded to, producing enlargements and tubercles where such derangements are seated, we not uncommonly find, upon dissection, an engorged state of the gall-bladder, with great distention of the hepatic ducts. In these cases, the bile contained in the gall-bladder is generally dark green, of a thick consistence, and extremely viscid; at other times it is almost as black as tar, and scarcely can be made to pass through the ducts. Occasionally the bile accumulated in the gall-bladder is of a straw colour and gelatinous consistence, and with difficulty can be squeezed through the cystic duct. In many instances, wherein a loaded state of the gall-bladder (such as is now pointed out) has been observed, in dissections of those who have died either of fever, dysentery, icterus, or of hepatitis itself, the ducts have been more or less diseased. In some cases they seem to have been spasmodically contracted; in others, they have been almost impervious, most probably from previous inflammation. In other instances they seemed to have been only plugged up by the thick and viscid nature of the secretion; and, in a few cases, lymph seemed to have been effused from the internal surface of the inflamed canal, and to have obstructed the passage of bile. These derangements, as far as my own observation goes, have generally been confined to the cystic duct." (P. 428—431.)

### *Of the use of Calomel in acute Dysentery.*

In this disease, scruple doses of calomel are recommended at night, with a purgative draught in the morning; in addition to which, it is frequently necessary to give the medicine during the day, that it may operate "through the medium of a partial absorption:" for which purpose, three grains of it may be given, with three or four of ipecacuanha and one of opium, ter die.

### *Of the Use of Calomel in chronic Dysentery and Diarrhœa.*

When the tongue is coated and foul, the evacuations scanty and difficult, with blood or mucus, we are advised to give "a scruple of calomel with two grains of opium, or ten grains of calomel with five of antimonial powder and one of opium," at night, and an aperient next morning. If these means fail on their first exhibition, they are nevertheless to be continued, but not to the exclusion of other means, as the warm bath and antimonials, so as to determine to the surface, and emollient and anodyne injections. When the motions have assumed a healthier character, an alterative treatment is to be adopted.

*Of the Employment of Calomel in Cholera.*

In this complaint, calomel is to be employed to fulfil the first and second general indications pointed out in speaking of fever.

Our author next proceeds to make some observations on the exhibition of calomel in the *diseases of children*, in which we find the same general plan recommended of giving the medicine in large doses, with long intervals, instead of small doses frequently repeated, as is more usually done. In *constipation, worms, marasmus, convulsions, acute and chronic hydrocephalus, croup, chorea, eruptive and other fevers*, calomel is regarded as a remedy of great power, when exhibited in full doses of ten or fifteen grains, and followed up by purgatives next morning. This, in fact, is the leading principle which characterises the work; and we are perfectly satisfied that Mr. Annesley is correct in supposing that the method, too frequently adopted in this country of treating almost all the diseases of children with doses of calomel frequently repeated and long continued, is erroneous in theory, and injurious in practice. Yet we do not entirely agree with him in regarding his method as exclusively preferable: for example, in croup, we think it better to give the remedy very frequently, so as to bring on mercurial action, because we have observed that mucous membranes do not so readily throw out lymph if the system be brought under the action of calomel, and this, not its effect upon the bowels, is the indication which we feel anxious to fulfil.

Mr. Annesley concludes his interesting work (which is incomparably better *got up* than medical works usually are,) with the following general remarks:—

"The prejudices which exist respecting the exhibition of calomel, have led me to enter more fully into the subject of its use in diseases, than may appear in many to have been necessary; but I conceive that these prejudices have arisen from the inefficient and hurtful manner in which it has been usually prescribed during the preceding half century, and from the views which have guided practitioners in exhibiting it. There has been too general a desire to produce the constitutional effects of this mineral, and too little attention paid to its operation as a purgative, when given in large doses; and to its influence upon the secretions, the functions of the bowels, and the condition of the stools, when exhibited with the intention of producing cathartic effects. And, even when it has avowedly been given as a purgative, it has usually been prescribed in small, irritating, and inefficient doses; and practitioners have generally contented themselves with resorting to it once or twice, or at most occasionally; and hence they have had no opportunity of experiencing the effects arising from its more protracted use, when given in large doses, and alternating it with purgative or aperient draughts, and such other remedies as the nature of the disease, and the circumstances of particular cases, may have required.

"Erroneous notions respecting the operation of calomel have also been long entertained, and a timid mode of exhibiting it has been the consequence. The dose of the medicine has been assigned by authors of modern Dispensatories, and other writers, with a precision and a limitation which were by no means requisite; and those who merely followed the recommendation of others, without thinking and acting for themselves, either blindly adopted the same practice, or, if they did attempt to modify it, proceeded no further than to exhibit the medicine more frequently: and thus they actually resorted to a more hurtful mode of prescribing it than any other they could have pursued.

"The experiments which I have detailed, but, still more decidedly, my extensive experience of the effects of large doses of the remedy, when given in the way I have described, show its propriety; but even this mode, although the safest which can be adopted, requires judgment and tact in the practitioner for its efficacious employment. The empirical and indiscriminate use of the best medicines, and the inappropriate application of the most efficient practice, have been the frequent causes of their unmerited neglect, and of the most erroneous notions respecting them: causes, however, which can operate only for a time, and which will gradually disappear before the more general diffusion of professional science, and a more accurate and enterprising spirit of inquiry." (P. 462—464.)

---

### DIVISION II.

### FOREIGN.

Art. II.—*Mêmoires sur la Nature et le Traitement de plusieurs Maladies.* Par M. le Baron Portal, premier Medecin du Roi; Chevalier de l'Ordre de St. Michel; Officier de l'Ordre Royal de la Legion d'Honneur; Membre de l'Institut (Academie Royale des Sciences); President d'Honneur perpetuel de l'Academie Royale de Medecine, du Cercle Medical, et de la Societé de Medecine pratique de Paris; Membre du Conseil general des Hospitaux et Hospices; Professeur de Medecine au College Royal de France, d'Anatomie au Jardin du Roi, &c. &c.—Bertrand, Libraire, Paris, 1825. Pp. xxi. 480.

*Memoirs on the Nature and Treatment of several Diseases.* By M. Baron Portal, principal Physician to the King; Knight of the Order of St. Michael; Officer of the Royal Order of the Legion of Honour; Member of the Institute; honorary perpetual President of the Royal Academy of Medicine, &c. &c.

It is not without some compunctious visitings of conscience, that we, who profess to be yet untouched by the hand of time, presume to criticise the labours of the veteran, the title of whose work stands at the head of this article. Baron Portal has been a public teacher of his profession for a period of nearly sixty years; he has published numerous works,—indeed, there is scarcely a medical subject which he has not illustrated; and now, at an age when most men are gone to their last habitation, or are content to repose under the shade of their laurels, he again appears before the public as an author. But extremes meet; and, without more particularly alluding to the old proverb, which would make the Baron very much our junior, we would venture to remark, that it is lamentable to see too many men on this side of the water, the decay of whose intellectual faculties is fully commensurate with that of their physical powers, still retaining all the eagerness for employment, all the ardour for professional or literary distinctions, which characterise, and honourably characterise, the young and rising practitioner. This we say is lamentable, because it too often destroys the effect of the well-earned reputation of former years,—it renders that old age ridiculous, which in itself ought to be, and is when employed with dignity, most respectable and venerable; and it is too often traceable to the indulgence of one of the meanest propensities of our nature, the love of gain. There are, in our estimation, many motives that ought to induce the aged practitioner to be silent, but very few to induce him to write. We by no means wish to insinuate that all, or even the greater number, of the above remarks are applicable to the author before us: at least, it is our intention to let our readers judge for themselves; though we are free to confess that some of the practice which we find recommended in the progress of the work does appear to us rather strange, and not at all reconcileable to our English notions, even after we have made an allowance for difference of climate, constitution, habits of living, &c.

The present volume of M. Portal's Memoirs, which is the fifth, contains essays on various subjects: the first consists of observations upon Typhoid, or pernicious Remittent and Intermittent Fevers;—the second memoir treats of those Inflammations of the Intestines which succeed to diseases of the liver;—the third, which in fact occupies above half the book, is an essay on what the Baron calls *Pneumatie*, for which we have no corresponding term, the nearest translation being Emphysema. The word pneumatie is, in fact, one of our author's coining; and he introduces it as explaining those diseases arising from the formation or development of different gases in the living body; in the same way that Hydropsie is applied to collections of fluid. He, therefore, subdivides pneumatie into several species, which we shall introduce to our readers' notice presently. Next to this long disquisition follows an essay on the mode of prescribing remedies, followed by the relation of several cases, in which the author's success was remarkable; and the volume finishes with the Oration pronounced by M. Portal, as president of the jury of the hospitals of Paris, for the nomination of what are called internal and external pupils in medicine and surgery.

We say nothing of the editor's advertisement, although it occupies one-and-twenty pages; since it only, in fact, details, somewhat more at large than we have done, the contents of the work itself.

We now proceed to the consideration of the first paper, containing observations on *typhoid, or pernicious remittent or intermittent fevers*, coming on, contrary to all expectation, during or after other diseases, and which have been cured by bark in substance.

M. Portal begins by stating the general belief, among medical men, in the efficacy of the cinchona in certain fevers and in gangrene, and the discordance of opinion as to its employment in many other complaints. To these points he directs our attention; but he previously gives us a few rules by which we may decide whether the use of the bark will be salutary or not; or, finally, whether it is likely to produce ill consequences. The chief of these aphorisms are—1st, that the cinchona is more efficacious in proportion as the fever approaches more perfectly to the intermittent type;—2d, that, instead of being salutary, it is generally hurtful where fever has no remissions; —3d, that, in the treatment of all fever, whether continued, remittent, or intermittent, there are circumstances which oblige us to administer the bark as soon as possible, without any previous preparation, such as the true typhus; whilst in other fevers the typhoid symptoms are so obscurely marked, with reference to others which are more apparent, that it is necessary to precede the exhibition of the cinchona either by vomits, purges, bleeding, blisters, or other remedies. In other circumstances, a fever may be considered rather beneficial than hurtful, and it might be dangerous to arrest it by the bark. With these distinctions we are not disposed to quarrel; but, if there is any justice in them, what are we to say of the following paragraph, which is illustrated by the relation of some very extraordinary cases; one or two of which we shall present to our readers.

After having appealed to the works of most of the celebrated physicians of France, England, and Italy, in confirmation of his views, our author adds—"It is not only in the treatment of continued, remittent, and intermittent fevers, regular or irregular, that the above-named celebrated physicians have happily employed the bark; they have also prescribed it, with admirable success, in the treatment of acute and chronic diseases, complicated with periodical accidents of different natures,

—such as pains, inflammations, comatose symptoms, of a greater or less degree of intensity, spasmodic, and even convulsive diseases; in fact, in a great number of cases where it has required great discernment to discover a febrile principle, sometimes even truly typhoid." (P. 6.) Now, the only comment we have to make upon this passage is, that, if this be true, there was no need of making any restrictions at all with regard to the employment of cinchona; since it will even succeed, as we shall presently see, under circumstances which would appear to forbid its exhibition entirely. We, therefore, at the risk of being thought tedious, proceed to translate the first case recorded, in illustration at once of M. Portal's views and our own.

M. De Lantier, author of the Voyage of Antenor, was attacked with jaundice, attended with enlargement of the liver, perceptible to the touch. The urine was high coloured, thick, and in small quantity. Some remedies had been prescribed for these symptoms, though without much benefit. The patient believing that he should be benefited by a residence at Corbeil with one of his friends, went there towards the end of September, after a very hot summer. Though rather better, he had a slight accession of fever every evening, which began with a trifling chill, followed by considerable heat, and sleeplessness during the night; with a little moisture of the skin towards morning, the heat of the skin being always strongly marked. The jaundice became intense, and œdema of the feet appeared, and made some progress. The fever, which had lasted some days, was one evening suddenly augmented in a very violent degree: the cold fit was longer and more intense; the hot stage was also aggravated; and the sweat, which succeeded towards morning, was copious, and followed by extreme weakness. The next day, the patient revived, and appeared better until the evening of the day following, when a new accession of fever took place. This was more violent than the preceding one, the hot stage being attended with delirium; and it terminated in abundant sweats and frightful weakness, amounting almost to syncope. The whole of the day, however, the patient appeared better, excepting that he felt weak. It was then thought proper to remove M. De Lantier to Paris; but he experienced a paroxysm of fever on the route, terminating in the same manner. The patient had remained nearly in a state of coma, replying only to questions by very vague and unconnected answers.

On the following morning, when visited by M. Portal, the patient was of a deeper yellow than when he went into the country; the hepatic region was more prominent, and harder; the pulse was very feeble; the skin moist; and the intellects were disturbed: nevertheless, it appeared to M. Portal that the patient was enjoying a remission of the fever, though in a state of such weakness as to make it probable that another paroxysm, which could not be retarded, would carry him off. Sinapisms were applied to the feet, together with the cinchona in the form of decoction, to be taken every two hours; and to each dose of which one drachm of the powder of bark was to be added. M. Portal took his leave, having given a fatal prognostic.

He then goes on to remark, that he should have ordered the bark with more confidence, had he been convinced that the fever which existed was really intermittent, for then he knew its success would have been complete; but he believed the disorder to be remittent, and that it was moreover complicated with disease of the liver. However, he conceived the danger was so imminent, that, having no other remedy for the fever but the bark, it would be easy afterwards to prescribe for the hepatic affection, provided he could save the patient from the febrile attack.

The next day there was but a slight return of the fever; and only two other and slighter paroxysms occurred. The use of the bark in powder, mixed in water, was prescribed for seven or eight days, in the cose of one drachm twice in the day; diminishing it gradually to a scruple three times, and afterwards once, in the day. The patient experienced such benefit from this treatment, that not only was the fever conquered, but the jaundice disappeared, and the size of the liver was considerably diminished. He drank the waters of Vichy for fifteen days, and was completely cured.

The only remark we shall make upon this case is, that, however fortunate the result of this treatment might have been, we conceive few Englishmen would have survived it.

Nine other cases, equally wonderful in their symptoms and success, follow the above relation; but we may be excused from entering more particularly into their details, since our author mentions the leading circumstances of them when detailing the results of his practice in these fevers, and which he does in substance as follows:—

Passing by his comments on the case we have detailed at length, we come to the second case, which, he says, affords a proof that the remittent fever attended with syncope *(fievre syncopale remittente)*, coming on in a patient labouring under anasarca, with jaundice and enlargement of the liver, recognisable by the touch, at the period of the cessation of the menses, was cured by the bark in large doses, mixed with water; to each dose of which one drachm of the acetate of ammonia *(spiritus mindereri)* was added, not on account of its febrifuge qualities, but as a diuretic.

The succeeding cases, until we arrive at the tenth, go to prove that the presence neither of rheumatism nor gout is sufficient reason for abstaining from the bark, in cases otherwise calling for its exhibition; and we have nothing to say against this general doctrine, though we might be disposed to contest its particular application in some of the examples the Baron has adduced; but, as they all recovered, (which is the goal to which every medical man strives to bring his patient,) we have not the courage to protest against a line of practice attended by such happy results. Nevertheless, we were somewhat startled to find that neither a threatening of hydrothorax, attended with the greatest difficulty of breathing in one case, nor a teasing cough, giving suspicion of an affection of the lungs, with something very like hectic fever in another, prevented our author from having immediate recourse to this remedy, which he not only prescribes by the mouth, but also administers in *lavemens*; and, whenever he intends to make sure work of it, it is generally combined with drachm doses of the spirit of mindererus.

M. Portal concludes this memoir by adroitly hoping that the new salts of bark may be found as efficacious as they are reported to be; but, with the caution of age, he withholds his belief until this shall be confirmed by a long experience of their effects.

The second memoir treats of *those inflammations of the intestines which succeed to diseases of the liver*. M. Portal remarks, that, although enteritis has been a disease acknowledged in all ages to be frequently met with, yet there were never known so many examples of it as at the present time. This he believes to arise in consequence of practitioners not making a proper distinction between those intestinal inflammations which are really idiopathic, and those which are consecutive upon diseases of other organs; a distinction which it is really useful to make, because they, in fact, require a different mode of treatment: to explain this difference is the object of the present paper.

The remarks which usher in the cases detailed by our author in illustration of this position, appear to be a covert attack upon the Brousaisian doctrines. The following paragraph is, we think, decisive upon this point:—"We fall," he says, "into great error if we deceive ourselves in this respect, (namely, whether the inflammation is immediately excited in the intestines, or secondarily from the lesions of other organs:) these errors are as pernicious as those which we commit when we attribute to the stomach diseases which reside in the liver." And again, in the next page:—"Besides," he continues, "since the time of Ferrein, the seat of fevers called gastric has been fixed in the stomach, although, in fact, they exist principally in the liver and the bile." (P. 54.)

Our author, then, in order to demonstrate the influence which the liver exercises over the intestines, refers, first, to the communication of nerves and vessels between those parts: secondly, to the juxta-position of the gall-bladder with the colon, as well as their union by duplicatures of the peritoneum; thirdly, to the communication of the liver, by means of the ductus coledochus, with the duodenum. How often it happens, he adds, that patients complain of violent pains in the umbilical region, the cause of which one would scarcely believe to exist in the liver, though that is the real seat, although there is neither jaundice nor any painful sensation in the region of that viscus; and such painful feelings have been accordingly attributed, falsely, to worms, to chronic inflammation of the bowels, or to other diseases which have been believed to have existed in them. In order to give our readers a specimen of our author's meaning, as well as his practice, we translate the following case.

A shopkeeper, in the street of St. Denis, had suffered frequently, and for a long time, such severe pains in the umbilical region, that an inflammation of the bowels was apprehended. She was about thirty years of age, of a strong constitution, but menstruated irregularly. Leeches were applied to the anus at one of the menstrual periods, which was imperfectly marked. She took some warm baths, together with some soap pills and light bitters, with infusion of orange and chamomile. She got well. After the lapse of a few months, however, she was assailed by fresh pains, and, without previous bleeding, aloetic pills and very heating drinks were recommended. The menses became suppressed, and all the signs of enteritis took place. Called to her assistance at this period, I caused her (says the Baron,) to be bled in the foot, and ordered relaxing drinks and warm baths, and afterwards the waters of Vichy. The menses re-appeared, the belly became relaxed; bilious stools ensued, and the patient was perfectly cured.

We must beg here to observe, that we do not see how the above case bears upon the point in question, or how it was well possible to make any mistake as to its real nature. The next case is still less obscure.

A man of a robust habit of body and bilious temperament, about forty years of age, applied to our author, complaining of violent pains in the belly, principally about the umbilical region. These pains had succeeded to nausea and vomiting of very bitter yellow matter, which had taken place the evening before, after a hearty dinner; though the same thing had frequently happened before, when he had not eaten much. The Baron examined the abdomen: the umbilical region was rather tumefied, the liver prominent under the edges of the ribs, and rather painful in the epigastrium. The patient's eyes were rather yellow, and his skin had the same tint. He said that he had often been more yellow; that his urine was sometimes as high coloured as blood, and his evacuations had been bilious. With these facts before him, the Baron pronounced his disease to be in the liver, and that the pains in the abdomen were only hepatic colic; but the patient could not be convinced, as he believed that he had taken poison. However, the Baron ordered him some leeches to the anus, some soap pills, with bitter extracts, warm baths, and the water of Vichy.

Our author heard no more of this patient for some months, when he returned to him labouring under the same symptoms, and confessing that he had employed many remedies, under the belief that he was threatened with inflammation of the intestines. He was ordered to have leeches applied to the anus, and not to the umbilical region, (this is the author's own distinction ;) the soap pills, with the bitter extracts, were again prescribed, only now they were made *un peu aloëtiques;* and afterwards the waters of Vichy. These remedies were attended with such success, that in about four months the patient returned to visit the Doctor, in a much improved state of health, as well as of mind. The same treatment was continued for some months longer, and he was radically cured.

These observations, which may at first sight appear trifling, become of importance when it is considered that we are here presenting our readers with the practice of one of the most eminent of the Parisian physicians, whose commentary upon the above case is this:—That there can be no doubt but that the pains in the bowels of this patient would have augmented, and that enteritis would have at length ensued, if he had not followed the Baron's advice; and even with more speed, if a stimulating plan had been adopted. To us the only wonder is, that the Baron's advice produced any effect at all.

After two or three other examples, equally precious, of enteritis following disease of the liver, our author proceeds to that which frequently succeeds to cholera morbus, or the iliac passion, as well as to some dysenteries, and which are owing to an alteration in the biliary secretion. Of this disease the Baron gives but one example, in the person of Mr. Madison, secretary to the English embassy at Paris at the peace of Amiens; but he does not tell us how we are to distinguish this fever from pure inflammation of the intestines ; nor does he say one word as to any peculiar mode of treating it.

With respect to that species of enteritis which sometimes comes on during the progress of typhous fever, and which he equally conceives to originate in a deranged condition of the liver, he observes that, though practitioners have neither remarked the particular symptoms during life, nor the changes that they might have observed in that organ after death, he does not doubt that, if they had observed them, they would have been perfectly convinced of the truth of his doctrine : and he affirms that, in persons who die of typhus, where the intestines appear inflamed, the liver is almost always swollen, hard in some parts, sometimes softer than natural, and of a very dark colour, its blood-vessels loaded, and the gall-bladder full of black bile ; although the small intestines, and even the stomach, may likewise be found containing a greater or less quantity. Nevertheless, he admits that, where the intestines are not at all diseased, the liver is found very much changed.

Our author has yet to speak of another form of enteritis connected with diseased liver, which attacks those persons who are affected with dilatation of the heart. The explanation of this effect is thus given :—The circulation of the blood in the vessels of the liver not being carried on freely in these individuals, because the hepatic veins cannot empty their contents into the right auricle, which itself contains too great a quantity, the liver is more and more overwhelmed with blood, and swells; at the same time the course of the bile is impeded, jaundice comes on; there is flatulence, abdominal pains especially in the navel,—and the pulse is hard and full. Every thing announces an enteritis, when, at the same time, the body often swells generally, but sometimes only the lower extremities, either from a collection of air or of water. One case of this kind is given, the only memorable point of which is, that the Baron was called into consultation with *seven* other physicians; and no wonder, therefore, that they were unable to decide upon the nature of the disease until the patient gave them an opportunity of ascertaining the fact of a very great dilatation of the ventricles of the heart, by dying, after a few months of ineffectual treatment.

The results to be obtained from this memoir are, 1st. That primitive inflammations of the intestines ought to be distinguished from consecutive, especially from those following disease of the liver, both on account of the prognostic to be given, and the treatment to be adopted. 2d. That these inflammations from affections of the liver are accompanied by symptoms which indicate those affections, such as jaundice, high-coloured urine, and others so obvious that we need not repeat them. 3dly. That those which are met with in typhus fever from the above cause are remarkable by the prostration of strength, by coma, often united with delirium, and by the state of the pulse, which is more unequal and less hard than in true enteritis. With regard to the mode of treating these diseases, we abstain from giving the Baron's rules; in the first place, because the case we have recorded explains sufficiently his method of practice, and again, because we do not think that the detail would afford either instruction or amusement to our readers.

The subject of what Mr. Portal calls Pneumatie, or Collections of Gas, occupies no less than 267 pages of the volume, and it commences with General Remarks upon Gases. These are ushered in by the following announcement:—"All the parts of our bodies contain gas or air more or less elastic, and other fluids, which do not possess the same degree of elasticity. In the healthy state, these fluids fulfil offices which are necessary to our well-being; in a state of disease, they may occasion different ill effects, according as they are vitiated either in quantity or quality."

We are then informed, that gases are divisible into three sections—1. Those that are respirable; 2. Those that are merely non-respirable; 3. And those that are deleterious, either by the irritation they occasion, or from any other cause. Upon these different gases, our author makes a few very general remarks, which are not worth repeating, and then proceeds thus: "To convince ourselves that in the healthy state gases exist in the mass of our fluids, it is sufficient to take away from a living animal a portion of a vessel filled with blood, after having tied both extremities, and to place it under the receiver of an air-pump; and, in proportion as the air is exhausted, it will be seen to tumefy. This can only be attributed to a diminution in the pressure of the atmosphere, so that the air contained in the vessel, being no longer compressed, is augmented in volume; its globules, which were scarcely apparent, unite one to the other; from whence it results, that the red globules of the blood are separated from each other, and, then troubling the circulation more and more, they at length change their nature, and are more or less disposed to be converted into water (page 96): the result of this is said to be a slower circulation of the mass of the fluids, from which numerous diseases, and even death itself, ensue. How many apoplexies, epilepsies, palsies, fevers, and other diseases, (exclaims our author,) which appear to us to differ in their symptoms, are nevertheless the inevitable consequence of the above cause; for it is not to be doubted that the air more or less vitiated penetrates, or is developed, much oftener than is thought of, in our vessels, and causes divers diseases of greater or less severity. These gases penetrate our bodies by means of respiration, with our aliments, or by the skin; and in a state of health, after having fulfilled those uses in the animal economy prescribed by nature, they are exhaled either in expiration, or by the excretory pores of the skin, as well as in the other excretions.

We now arrive at our author's reasons for preferring the word Pneumatie to Emphysema, and then have the following definition of the disease:—It consists in a slight soft elastic swelling, formed by gas or air collected in the cellular tissue of every part of the body, in its different vessels and cavities; if the disease is exterior, the swelling quickly returns, after compression, to render a sound not unlike that of a drum, whence arises the term tympanitis, given to one species. It may be either general or partial, and differs from dropsy in not affording the sense of weight usually felt in that complaint, as well as not preserving the impression made by pressing upon the swelling for the same length of time: however, gaseous swellings are sometimes extremely hard, and occasionally they disappear by the mere efforts of nature. They have been mistaken also for abscess, and opened with the lancet; they have, from their pulsation, been also mistaken for aneurism; and numerous diseases, says Mr. Portal, attended with coma and convulsions, have been caused by gases, which have been recognized, upon opening of bodies, either in the brain, the spinal marrow, the lungs, heart, stomach, liver, &c. the nature of which was not understood. For examples of this kind we are referred to Morgagni, Lieutaud, &c. Of the causes of this class of diseases not a word is said in any way satisfactory, but the following species are enumerated :—

1. The Pneumatie, which proceeds from excess or deficiency of evacuations.

2. From fevers.

3. From plethora.

4. From inflammation, and its consequences.

5. From different morbid affections, with or without fever.

6. From excessive eating, from bad food, or poisons, &c.

7. From pains; such as dentition, worms, wounds, contusions, &c.

8. From swellings or tumors, and different obstructions, and sometimes terminating in suppuration.

9. From what precedes, accompanies, or succeeds to convulsive, spasmodic, comatose, or paralytic attacks.

And 10. From artifice or imposition.

From this long list of species, it will be perceived that we have work enough upon our hands, without indulging much in our critical vein; nevertheless, whilst we have the opportunity, we may as well here observe, that if Pneumatie is likely to follow all the different conditions which our author says will frequently produce it, we scarcely know who is to escape. Another more serious defect attaches to the first species, for we are not informed as to the mode of recognising the disease, nor, excepting in one instance, how we are to treat it; so that we are left rather in a worse condition than if we had remained in happy ignorance of the existence of these formidable complaints. But our author shall speak for himself: nothing is more common, he observes, than to see gaseous swellings arise in those who have suffered from considerable evacuations, either by the skin, by urine, or stool, or even from salivation, or leuchorœa, but more particularly after great losses of blood, though it is not always possible to appreciate the danger from the mere quantity of blood lost, as so much will depend upon the constitution of the individual. Those labouring under phthisis, we are told, are only affected with Pneumatie after severe sweats, or colloquative diarrhœa. These emphysematous swellings also, according to M. Portal, are no less common after wounds, after excessive loss of blood, even from the application of too many leeches; and we are especially told, that after bleeding, too often repeated, collections of gas have been met with in the brain, and other organs. Finally, these gaseous swellings also appear with greater or less rapidity in the veins, after other evacuations, by vomit, purgings, &c. ; and it disappears only in proportion as the losses, with regard to quantity, are repaired either by the efforts of nature or the assistance of art. This remark leads our author to dilate a little upon the necessity of giving nourishing diet and appropriate tonics in those persons exhausted by excessive evacuations; and thus ends the chapter on Pneumatie from excess of evacuations: so that, as we before said, we are neither told how to recognize it when we find it, and still less how to treat it. But we believe, from the internal evidence afforded by some of the cases recorded, that, after all, a great majority of these gaseous swellings are nothing more or less than œdema.

Next in order comes an enumeration of the cases of Pneumatie produced by defective evacuations, such as those of the skin, urine, and fæces ; the suppression of the lochiæ in women in child-bed; that of the hæmorrhoidal flux, or of habitual bleedings at the nose, or of ptyalism. The same thing also happens from the cessation of morbid discharges, such as occur in diarrhœa, dysentery, and even of the bile itself, which, independently of its uses in digestion, acts the part (according to M. Portal) of preventing and restraining the expansion of gases so frequent in those persons in whom the bile does not flow in sufficient quantity. In addition to all these causes of the disease, we must not omit to mention the suppression of certain cutaneous eruptions, of the discharge from some kinds of ulcers, which have the effect of issues in preserving the health. Among all these varieties, the Pneumatie from suppressed transpiration, we are told, is the most common: sometimes there is only a puffiness of the face, hands, or feet; sometimes it affects one-half of the body, either longitudinally or transversely ; sometimes it attacks the whole body, exteriorly as well as interiorly. Of the above effects, M. Portal offers some examples, which we have been in the habit of considering as œdema, especially those swellings sometimes consequent upon the application of blisters ; and we are still of the same opinion, —for our author himself, after saying that they are *veritablement* formed of air, observes that they are very often replaced by œdema; and in another place, in detailing the case of a lady affected with Pneumatie from using cosmetic baths, we are informed that the termination of the disorder was a fatal dropsy.

Now, respecting the treatment of these cases, our author gets rid of this part of his subject very happily, by observing, that we must restore those evacuations which have been suppressed, and that this cannot be done unless we know how to prescribe appropriate remedies; and, finally, that it would be possible to make most important remarks upon this subject, which however he dismisses, most provokingly, without making any remarks at all.

The length to which this article has already extended, obliges us to notice some of the succeeding species of Pneumatie very cursorily. In that which is said to arise from fevers, we are told that it may occur in typhus, in remittent or intermittent fevers: of this latter species, two examples are recorded. On emphysema from plethora, M. Portal does not say much; but he relates one or two cases, which appear to us to be misplaced; take the following example:—

The Abbè Medale was seized with a general Pneumatie: he was of a strong constitution, fifty-five years of age, and very irritable. He complained of severe colics, for which carminatives had been prescribed, not only without success, but actually with an aggravation of the complaint; the abdomen swelled suddenly, and was sonorous when struck upon. The patient also complained of hearing noises in the belly when he moved, or even upon breathing deeply; the epigastric region was especially tumefied after the slightest repast, and the breathing was so disturbed, that suffocation was dreaded. It was thought that there was air in the chest. In this state, M. Portal was called in, who was apprehensive of anasarca, as the ankles were rather œdematous, but the urine was not diminished in quantity, and was free from sediment. Instead of warm diuretics, which the Abbé was taking, our author, as usual, ordered leeches to the anus, with chicken-broth, in which the leaves of pillitory of the wall were lightly boiled, and some cheroil afterwards infused, with the addition of ten grains of nitre to a chopine of the liquid. Under this treatment the patient was reduced exceedingly; then he drank asses milk, and got well, contrary to all expectation.

We do not know how our readers may feel, but we are heartily tired of recording such puerilities; and, with regard to the above case, we cannot help suspecting that this poor oppressed Abbé's case had better have been classed among those cases of Pneumatie arising from excess of eating. The account puts us very much in mind of a *bon vivant*, puffed up with a mass of ill-digested food, overwhelmed with flatulence, and most probably having the large intestines crowded with fæcal matter. As to the threatened danger of suffocation, who has not witnessed something approaching to this from constipated bowels, combined with dyspepsia?

Whether we shall have the courage to encounter M. Portal in a subsequent Number, depends upon too many contingencies to permit us to pledge ourselves either one way or the other.

